# Systems biology applied to vaccine and immunotherapy development

**DOI:** 10.1186/1752-0509-5-146

**Published:** 2011-09-20

**Authors:** Luigi Buonaguro, Ena Wang, Maria Lina Tornesello, Franco M Buonaguro, Francesco M Marincola

**Affiliations:** 1 Molecular Biology and Viral Oncology, Dept of Experimental Oncology, Istituto Nazionale Tumori "Fond Pascale", Via Mariano Semmola 142, 80131 Napoli, Italy; 2 Infectious Disease and Immunogenetics Section (IDIS), Department of Transfusion Medicine, Clinical Center, and trans-NIH Center for Human Immunology, National Institutes of Health Bethesda, Maryland, USA

## Abstract

Immunotherapies, including vaccines, represent a potent tool to prevent or contain disease with high morbidity or mortality such as infections and cancer. However, despite their widespread use, we still have a limited understanding of the mechanisms underlying the induction of protective immune responses.

Immunity is made of a multifaceted set of integrated responses involving a dynamic interaction of thousands of molecules; among those is a growing appreciation for the role the innate immunity (i.e. pathogen recognition receptors - PRRs) plays in determining the nature and duration (immune memory) of adaptive T and B cell immunity. The complex network of interactions between immune manipulation of the host (immunotherapy) on one side and innate and adaptive responses on the other might be fully understood only employing the global level of investigation provided by systems biology.

In this framework, the advancement of high-throughput technologies, together with the extensive identification of new genes, proteins and other biomolecules in the "omics" era, facilitate large-scale biological measurements. Moreover, recent development of new computational tools enables the comprehensive and quantitative analysis of the interactions between all of the components of immunity over time.

Here, we review recent progress in using systems biology to study and evaluate immunotherapy and vaccine strategies for infectious and neoplastic diseases. Multi-parametric data provide novel and often unsuspected mechanistic insights while enabling the identification of common immune signatures relevant to human investigation such as the prediction of immune responsiveness that could lead to the improvement of the design of future immunotherapy trials. Thus, the paradigm switch from "empirical" to "knowledge-based" conduct of medicine and immunotherapy in particular, leading to patient-tailored treatment.

## Review

### Cross-talks between innate and adaptive immune systems

Pathogen recognition receptors (PRRs) detect foreign antigens in the form of living pathogen or vaccine [1,2] activating specific signaling pathways that drive biological and immunological responses. Among the PRRs, Toll-like receptors (TLRs) are widely present on innate immune cells (including DCs, macrophages, mast cells, neutrophils), endothelial cells and fibroblasts, and their expression is regulated by several factors, including foreign antigens, vaccines and cytokines [3-6].

Twelve members of the TLR family have been identified in mammals to date, and different TLRs are expressed extra- or intracellularly. In particular, TLRs 1, 2, 4, 5, and 6 are expressed on the cell surface, whereas TLR3, 7, 8, and 9 are found almost exclusively in intracellular compartments such as endosomes [1,2]. Each member of the TLR family recognizes a specific structural component of pathogens [1,7-19]. In addition to TLRs, other important families of PRRs are plasma-membrane and cytoplasmic receptors, including the C type lectins, which recognize a range of microbial stimuli from pathogens such as HIV, HCV, Helicobacter pylori, and Mycobacterium tuberculosis [20,21], and NOD proteins, which recognize components of intracellular bacteria [22].

The interaction between PRRs and foreign antigens expressed by the vaccine triggers a downstream signaling cascade leading to several cellular processes, including production of proinflammatory cytokines and chemokines. In particular, TLR activation induces a signal cascade via several intermediates, whose endpoint is the activation of transcription factors turning on the expression of inflammatory cytokine genes, such as TNF-β, IL-6, IL-1β, and IL-12 [1,23].

Subsequent to the TLR-ligand interaction, APCs (mainly DCs) uptake and process vaccine antigens to be exposed on cell membrane surface in association to major histocompatibility complex (MHC) molecules for efficient presentation to adaptive immune cells [24]. DCs undergo full activation and maturation, characterized by the increased expression of co-stimulatory molecules (CD40, CD80, CD86) and production of chemokines (TNFα, RANTES, MIP1α, MIP1β). Activated and matured DCs migrate to the regional lymph node, where they provide antigen-specific activation as well as co-stimulatory signals to naïve T cells and possibly B cells, bridging innate with adaptive immunity [25-27].

Engaged naïve T cells undergo clonal expansion and differentiation into effector CD4+ T helper cells or CD8+ cytotoxic T lymphocytes (CTL). CD4+ T-helper cells can be directed into a Th1, Th2 or T-Reg polarization upon direct contact with antigen-bearing APCs and induction by specific cytokines. The polarized T-helper cells can antagonize each other's actions and will ultimately lead the adaptive immune system toward either a cellular (Th1), a humoral (Th2) or a tolerance (T-Reg) response [28-33]. A subset of polarized activated effector T cells will further differentiate into long-lasting memory cells to readily induce an immune response at subsequent encounters with the same antigen [34].

In such framework, a successful vaccine must be effective in activating PRRs. However, the degree of TLRs engagement has been studied only for few licensed vaccines, including Bacillus Calmette-Guerin (BCG) [35,36], Haemophilus influenzae type b (HiB) [37] and live attenuated yellow fever vaccines [38]. For other licensed vaccines, although the engagement of TLRs has not been documented, it could be inferred that live attenuated vaccines activate innate immunity following the same pathway induced by the corresponding native fully replicating virus. On the contrary, inactivated or subunit vaccines may be not efficient in activating the innate system, requiring the addition of an adjuvant in the formulation to improve their immunogenicity. Among the very few adjuvants approved for human use, only the Monophosphoryl Lipid A (MPL) is known to engage a TLR (TLR-4), being a non-toxic derivative of the lipopolysaccharide (LPS) of Salmonella Minnesota [39,40], but many new vaccine adjuvants are under development and evaluated in clinical trials, whose mechanism of action is mediated by TLRs [41-46]. Recently, we reviewed the differential transcriptional activation of monocyte-derived DCs following distinct and frequently applied maturation strategies and their strong effect on Th-polarization. This review clearly demonstrated that the stimulus applied for DC maturation can affect dramatically the global expression pattern of these cells and, consequently, their *in vitro *and *in vivo *function [47].

#### Vaccine development

The goal of a successful vaccine is to induce long-term protective immunity based on the generation of an antigen-specific immunological memory. This is achieved via several levels of cross-talks between the innate (antigen-presenting cells - APCs) and adaptive (T and B lymphocytes) immune systems involving both cell-to-cell contact and/or soluble factors (i.e. cytokines and chemokines).

Most of the current successful vaccines are based on live attenuated or inactivated pathogens which show distinctive biological and immunological characteristics. The live attenuated vaccines are viruses with a limited replication in the vaccinated host, carrying the native pathogen-associated molecular signals - PAMS (i.e.: viral genetic material) which bind the pathogen recognition receptors - bind PRRs and trigger the activation of the innate immune system. Such attenuated viruses mimic a natural infection and spread to multiple host immune organs or tissues, eliciting immune responses similar to those induced by fully-replicative pathogens, which are often effective after a single administration [48]. The major drawback of such strategy is the mild-to-severe adverse effects as consequence of the limited replication in the vaccine recipients. The inactivated vaccines are viruses which cannot replicate due to irreversible damage of genetic material induced by heat or chemical treatment. Although safer than attenuated vaccines, they are generally less effective and require multiple administrations to boost the immune response documented for instance by enhancement of antibody titers over time. The inactivated vaccines are made of either whole virus or subunits (i.e. viral proteins) relevant to the conferring of protective immunity.

In the last years, also for safety reasons, alternative non-replicating vaccine strategy, including recombinant proteins, synthetic peptides, DNA, particulate structures (i.e.: Virus-Like Particles) have been developed [49]. However, despite relevant safety advantages, such vaccine strategies are not always effectively processed by antigen presenting cells and presented to the adaptive immune system, lacking "danger" signals necessary to trigger activation of the innate system and, downstream, of the adaptive immune response [50].

#### The role of active specific immunization (vaccines) among immunotherapy strategies

Immunotherapy is a broad term that encompasses any manipulation of the immune response to elicit clearance of unwanted conditions such as infections, cancer, autoimmunity and rejection of heterologous organs. The purposes are somewhat opposite in some compared to other conditions as in some cases immune mediated, tissue-specific destruction is desired to clear the organism of cancer cells or cells infected with pathogens while in other cases efforts are made to dampen the same immune mechanisms that are actively causing unwanted destruction of tissues such is the case for autoimmunity, transplant rejection and graft versus host disease; we have recently shown that although the mechanisms leading to each of this pathological states or their resolution may be different, the final mechanism leading to tissue destruction is quite similar and involves the activation of a limited number of immune effector genes which we have called the immunologic constant of rejection (ICR) [51]. Thus, the role of immunotherapy is to enhance the chances of reaching full activation of ICR genes on one hand and reduce it on the other. It has become clear, thanks to transcriptional analyses that activation of an effector immune response is a multifactorial event that includes the activation of all arms of effector immunity including both innate and adaptive mechanisms and that neither alone is sufficient to induce tissue specific rejection [51,52]. Thus, it is likely that enhancement of one or another aspect of immunity by isolated immunotherapy strategies will be unlikely to achieve clearance of disease and combined strategies should be sought. Currently, immunotherapy strategies could be characterized by four major approaches: those aiming at the systemic and non-specific activation of immune cells (active immunotherapy), those aiming at the activation of specific antigen recognition pathways by T or B cells (active-specific immunotherapy), those in which cells deemed to bear important effector mechanisms (either antigen specific or non-specific) are expanded ex vivo and transfused in large number is patients (adoptive immunotherapy) and, finally, those in which effector molecules such as antibodies are transfused into patients (passive immunotherapy). While each one of these approaches has shown marginal benefit in the treatment of chronic infections and cancer, none of them alone has been dramatically effective in clearing disease and enhancing survival. In this context, active specific immunization with vaccines has the limited purpose of enhancing antigen-specific recognition by immune cells; interestingly, this has not been very successful in inducing dramatic clinical responses both in viral and in cancer systems. Recently, however, several clinical trials in which active specific immunization has been tested as a modality of treatment for cancer in large randomized trials have shown that survival is modestly but significantly enhanced compared to control groups treated with standard therapy. These studies provide a proof of principle that vaccination is probably enhancing the ability of the immune system to specifically recognize diseased tissues and control their growth. Yet, as we postulated in the ICR paper, the mechanisms leading to control of growth and presumably complete clearance of abnormal cells expand from the simple T or B cell interactions with their target to the activation in the diseased organ of an acute inflammatory process, triggered by the production of pro-inflammatory chemokines and lymphokines by antigen recognizing cells which can progressively recruit all arms of immune effector function [51].

#### Induction of adaptive immune response by vaccines

Most of the successful vaccines developed to date induce a long-lasting protective humoral adaptive immune response, eliciting the production of neutralizing or opsonizing antibodies [53]. However, such vaccines are effective mainly against pathogens characterized either by a limited and stable range of serotypes (i.e. smallpox, rubella, polio) or by seasonal serotypes (influenza). In the latter case, however, flu vaccine must be manufactured and administered each year according to the circulating seasonal variant.

However, in many cases the humoral antibody response by itself is not sufficient to protect against pathogens and, in the last years, the development of vaccines able to elicit also an effective cellular adaptive immune response has become a priority. In fact, if antibody-based vaccines provide prevention and protection from infection, T-cell-based vaccines may be relevant in controlling established chronic infections, such as HCV and HIV viruses [54-57], or a cancer [58-60]. This is particularly the case for chronic infections in which viruses are harbored within the cellular compartment. In that case, antibodies have no ability to recognize their target as they cannot cross the cell membrane and only immune cells with cytotoxic function can kill the infected cells and stop the perpetuation of the infectious process.

Regardless of the humoral or cellular immunity elicited by vaccines, relevant markers are needed to evaluate the vaccine efficacy and/or to optimize its development. Concerning the humoral immune response, a direct relationship between protective effect and serum titers of antibodies with high specificity and affinity, measured in ELISA or in neutralization has been widely accepted [61]. However, in few cases, other markers are considered better surrogates of protection, such as mucosal IgA titers for Rotavirus vaccine [62] or CD4+ T cell responses for Zoster vaccine [63], although in such cases no threshold levels for protection have been identified.

On the contrary, in situations where the humoral antibody response is not the protective arm of the immune response, specific parameters need to be validated to assess the relationship between protection and levels as well as type of cellular immunity. Indeed, a consensus has not been reached yet and different experimental models have been taken into account effector CD8+ cytotoxic T cell activity [64,65], perforin expression [66-68] or cytokine production [69,70].

#### Systems biology in vaccine development

**Figure 1:**
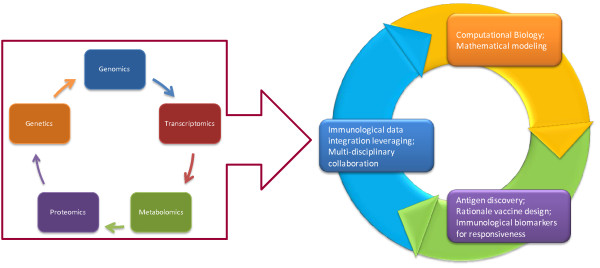
**Systems biology approaches for vaccine studies interactions and implications on translational research**.

The need for clear immunological markers to predict and evaluate the immunogenicity of vaccines and to optimize vaccine formulation critically exemplifies the usefulness of systems biology approaches. A great example was recently provided by a phase III randomized study in which a MAGE-A3 peptide was used to treat patients with advanced non-small-cell lung cancer or melanoma; this study not only demonstrated that the vaccine improved survival in either disease but also, by applying global transcriptional analysis to pre-treatment samples, the investigators identified signatures that clearly predict responsiveness to treatment both by immunological assessment and clinical benefit [71]. Indeed, the advancement of high-throughput technologies, together with the extensive identification of new genes, proteins and other biomolecules in the "omics" era, has facilitated large-scale biological measurements. The new experimental paradigm of systems biology aims to consider a biological system as not just a set of distinct elements, but rather as a complex product of the interactions among these elements and their relationship with the surrounding environment (Figure 1). Importantly, there are two major approaches to systems biology: a top down view in which potential permutations predicted by experimentally formed knowledge are predicted with a deductive approach; conversely, and inductive approach is often applied in clinical research following a bottom up approach; in this case, the global picture associated with a specific biological occurrence are "photographed" using high throughput technology and the reasons for their occurrences are then hypothesized using a reverse engineering approach [72]. We contend that this evidence based approach is a necessary preliminary step to focus the aim of future investigations because it identifies concept relevant to human physiopathology during the natural course of the disease or it response to treatment.

Despite the increasing use of such approaches in oncology [73-75], autoimmunity and infection [76,77] for the identification of prognostic or predictive biomarkers, systems biology has been only recently applied to vaccinology though the number of information are steadily increasing [78-80]. Each of the available high throughput methodologies have been employed to study the immune response induced by vaccines and a short description is reported here (Table 1).

**Table 1:** Examples of Systems Biology applied to vaccinology

Strategy	Vaccine model	Ref
**Microarray**	Yellow fever	[88,89]

**Microarray**	HIV	[90,93]

**Microarray**	Melanoma	[112,113]

**Microarray**	NSCLC	[114]

**SNPs**	Measle	[120,122]

**SNPs**	Rubella	[123]

**SNPs**	Pertussis	[126,127]

**SNPs**	Influenza	[130,131]

#### Transcriptomics

Transcriptomics enable the identification of set of genes and pathways differentially regulated in immune cells upon encounter with a foreign antigen or other non antigen-specific stimulation. DNA microarray technology has provided new insights into interactions between pathogens and innate immunity [81-85], which represent the background information for understanding and predicting the host response to vaccines. More recently, new powerful transcriptomics technologies have become widely available, including next-generation sequencing, exon and microRNA arrays [86,87].

#### Vaccines for infectious diseases

Gene 'signatures' in humans have been recently identified to predict immune responses to yellow fever vaccine (YF-17D) [88,89] and VLP-based HIV vaccine [90-93]. Transcriptional profile of early innate immune genes in PBMCs from vaccinated individuals showed that vaccination with YF-17D induced in most vaccine recipients a signature inclusive of genes involved in innate sensing of viruses and antiviral immunity. Among these, indeed, were observed genes encoding innate sensing receptors (i.e.: TLR7, RIG-I), transcription factors that regulate the expression of type I IFNs, IFN regulatory factor 7 (IRF7) and signal transducer and activator of transcription 1 (STAT1). A group of transcription factors, including IRF7, STAT1 and ETS2, were identified as key regulators of the early innate immune response to the YF-17D vaccine [88,89]. Furthermore, the enhanced transcription of several downstream genes that play critical roles in the maturation and differentiation of T cells, B cells, NK cells, and macrophages was observed [89].

Further bioinformatics approaches applied in a second YF-17D vaccine trial identified two genes - solute carrier family 2, member 6 (SLC2A6) and eukaryotic translation initiation factor 2 alpha kinase 4 (EIF2AK4) - that did correlate (with 90% accuracy) with the magnitude of antigen specific CD8+ T cell responses and antibody titers. In particular, EIF2AK4 regulates protein synthesis in response to environmental stresses by phosphorylating elongation initiation factor 2α (eIF2α) [94,95]. Indeed, YF-17D vaccination induced the phosphorylation of eIF2α as well as the formation of stress granules, and other genes involved in the stress response pathway correlated with the CD8+ T cell response [88].

The TnF receptor superfamily, receptor 17 (TnFRSF17), which is a receptor for B cell-activating factor (BAFF), was shown to be a key gene in the predictive signatures. BAFF, indeed, is thought to optimize B cell responses to B cell receptor- and TLR-dependent signaling [96,97].

Similar studies have been performed using a baculovirus-expressed HIV-VLPs developed in our laboratory [98]. Such HIV-VLPs, indeed, induced specific transcriptional profiles of genes involved in the morphological and functional changes characterizing innate and early adaptive immune response. This immune signature was observed in MDDCs [90] as well as in PBMCs from HIV-1 seronegative and seropositive subjects [93,99].

In particular, as described for the yellow fever live attenuated YF-17D vaccine, HIV-VLPs induced a molecular signature including several genes involved in innate sensing of viruses and antiviral immunity. Expression of proinflammatory mediators CXC-chemokine ligand 10 (CXCL-10) and interleukin-1α (IL-1α) genes were found upregulated. Similarly several genes were identified encoding innate sensing receptors (i.e.: TLR2), transcription factors that regulate the expression of type I IFNs, IFN regulatory factor 1 (IRF1) and signal transducer and activator of transcription 2 (STAT2). The gene signature predictive of both humoral and cellular adaptive immune response included several genes. The CD83 and CD28 genes indicate a strong activation of the Th2 development and B lymphocytes [100-102]. The TNF receptor superfamily, receptor 1B and 6B (TnFRSF1B and TnFRSF6B) are a marker for T and B cell activation (TnFRSF1B) [103] and resistance to the pro-apoptotic activity of the FAS-ligand (TnFRSF6B) [104]. The TNFSF9 is a T-cell activation marker [105,106] and the CD40 is one of the key players in activation of both humoral and cell-mediated immune responses [107,108].

**Figure 2:**
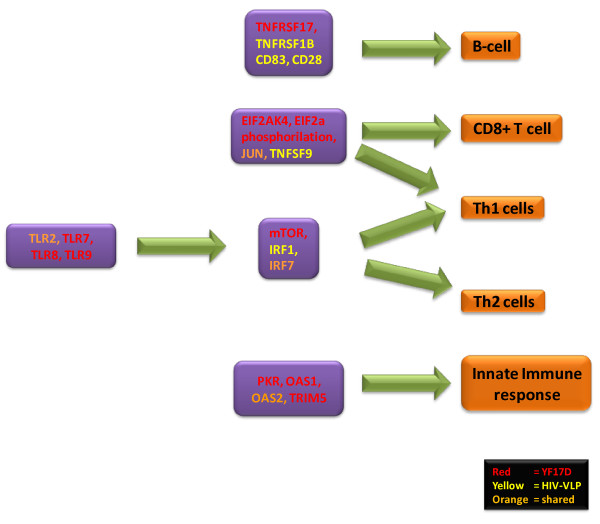
**Genes modulated by YF-17D and HIV-VLP vaccines identified by microarray analyses**.

These studies provide a global description of the innate and adaptive immune responses induced by two vaccines based on two different strategies, live attenuated (YF-17D) and non-replicating Virus-Like Particles (HIV-VLPs), identifying commonalities between the signatures induced by the two vaccines. Such results suggest the possible identification of specific shared predictive gene expression meta-signatures with a broad application in vaccinology (Figure 2).

In such studies, predictive signatures of response to vaccine have been evaluated using two independent classification methods, called classification to nearest centroid (ClaNC) and discriminant analysis via mixed integer programming (DAMIP) [88]. In particular, ClaNC has been previously shown to successfully develop predictive transcriptional cancer models [109], while the DAMIP classification model has proven to be a powerful supervised-learning classification approach in predicting various biomedical and bio-behavioral phenomena [110] and to produce superior classification accuracy when compared to traditional quadratic or linear discriminant analysis [111]. The DAMIP method was found to useful in determining correlates of vaccine efficacy for both T cell and B cell responses [88].

#### Vaccines for cancer

A selected subset of genes differentially expressed in melanoma metastases lesions have been identified, which are associated with better response to vaccines [112]. In particular, patients with a clinical response showed a gene transcriptional profile characteristic of an inflammatory tumor microenvironment, including chemokines and T-cell markers, that existed before the initiation of vaccination. A transcriptional profile involving similar set of genes was associated with better clinical outcome upon administration of MAGE-A3 protein vaccine [113]. Indeed, upregulation of CCL5, CXCL9 and CXCL10 chemokine genes within the melanoma metastases has been identified in patients with improved clinical outcome following vaccination, suggesting an effective intra-lesion recruitment of effector T cells [112].

Very interestingly, the same set of genes has been correlated to better outcome in subjects affected by non-small-cell lung carcinoma (NSCLC) and vaccinated with the same MAGE-A3 strategy [114].

Such results suggest that the pre-existent inflamed tumor microenvironment able to recruit activated T cells may represent a favorable prognostic factor for clinical response to cancer vaccines.

#### Genetic polymorphisms in innate immunity genes

Genetic polymorphisms can adversely affect expression of genes as well as proteins of the innate immune system and, consequently, host-pathogen interactions and molecular signaling. This represents an additional level of analysis to be included in the global evaluation of factors involved in the host response to foreign antigens, including vaccines [115-119].

##### Measle Vaccine

Associations between SNPs in TLRs 3, 4, 5 and 6 and the downstream intracellular signaling molecules, MyD88 and MD2, with variations in both antibody and cellular responses following measles vaccination have been recently described [120]. A SNP in the 3'UTR of TLR3 (rs5743305 at -976 bp of TLR promoter) has been identified demonstrating an association between heterozygous variant AT and low antibody as well as low lymphoproliferative responses in vaccinees. Similarly, the GA variant of a non-synonymous SNP also in the TLR3 gene was associated with lower antibody production.

Moreover, heterozygous variants for two non-synonymous SNPs (Gly299Asp and Ile399Thr) have been identified in the TLR4 gene and associated with higher IL-4 secretion to the measles vaccine strain [120]. Of note, the same two SNPs have been studied extensively in association with septic shock after infection with gram negative bacteria, premature birth, myocardial infarction and allograft rejection [121]. More recently, the major alleles of coding SNPs in the TLR2 (rs3804100) and TLR4 (rs5030710) genes have been associated with a dose-related increase or decrease in measles-specific antibodies, respectively [122]. Similarly, associations between SNPs in TLR5 and TLR6 genes and variations in IFN-γ secretion in response to measles virus stimulation have been identified, whose significance is still unclear [120].

Associations between SNPs in genes of intracellular signaling molecules associated with TLRs and the immune response to measles have been also investigated and a minor allele variant for a SNP in the 3'UTR of MyD88, the intracellular adaptor molecule that signals for most of the TLRs, was found to be associated with a lower antibody response to measles vaccine. Furthermore, several intronic SNPs in TLR and their associated intracellular molecule genes were significantly associated with variations in cellular immune responses to measles vaccine [120].

#### Rubella Vaccine

Also for rubella vaccination, associations between SNPs in TLR and the downstream intracellular signaling molecules with variations in both antibody and cellular responses have been recently described [123]. Polymorphisms in promoter and intronic regions of TLR3 and TLR4 genes have been found associated with rubella virus specific cytokine immune responses, such as IFN-γ, IL-2, TNF-α, and GM-CSF. In particular, two SNPs in the TLR3 gene appear to be significantly associated with lower rubella IFN-γ secretion in an allele dose-related manner. Of note, the promoter polymorphism (rs5743305, -8441 A > T) in the TLR3 gene, associated with rubella virus induced GM-CSF secretion, is the same SNP suggested to be a risk factor for lower antibody and low lymphoproliferative responses to measles vaccine [120]. This finding strongly suggests that rs5743305 in the TLR3 gene may play a role in viral immunity and may be a key control point for humoral and cellular immune responses to both measles and rubella vaccines.

The same study identified 22 associations between polymorphisms in promoter and intronic regions of vitamin A and vitamin D receptor genes and their downstream mediators of signaling with different immune response to rubella-specific cytokine. Since SNPs in the vitamin D receptor (VDR) genes have been associated also with protection from HIV-1 infection [124], it can be postulated that pro-inflammatory immune responses to viral infection or live viral vaccination are influenced by functional polymorphisms in the VDR gene.

Finally, associations of polymorphisms in the TRIM5 gene with variations in rubella virus-specific immune responses (TNF-α, GM-CSF and IL-2) have been observed, in concordance with recent findings on the role of the same TRIM5-gene SNPs in the immune response to retroviral (HIV-1) infection [125].

#### Pertussis Vaccine

The involvement of TLR4 in immunity to B. pertussis vaccine has been extensively shown and specific SNPs in the promoter region of the TLR4 gene influencing the antibody response to the pertussis (PT) vaccine have been identified [126,127]. The evidence of association was most consistent and strong for the SNPs in the TOLLIP gene, which showed association in three independent analyses. TOLLIP is a small protein that binds the activated IL-1 receptor type I (IL-1RI) complex, as well as TLR2 and TLR4 complexes, coordinating optimal signaling through IL-1RI and TLR4 [128,129].

Furthermore, associations of SNPs in TIRAP and TICAM1 genes and immune response to PT vaccine can be explained by the knowledge that these two factors belong to the Toll/Interleukin-1 receptor (TIR) domain-containing adaptors, also including MyD88, that modulate TLR signaling pathways. Furthermore, the signal transduction mediators of the Toll and IL-1 receptor (IL-1R) families, namely IRAK3 and IRAK4, showed evidence for association with immune response to PT vaccine.

#### Influenza vaccine

The role of HLA gene polymorphisms in the response to influenza vaccine has been evaluated and HLAA* 1101 (p = 0.0001) as well as A*6801 (p = 0.09) alleles were associated with higher median levels of antibody titers to influenza vaccine [130]. In the same study, also polymorphisms of cytokine and cytokine receptor genes were associated with humoral response to seasonal influenza vaccination. Previously, the increased frequency of HLADRB1* 0701 and the decreased frequency of HLA-DQB1*0603-9/14 was identified in individuals non-responders to the influenza subunit vaccine [131].

The correlation between specific gene polymorphisms and responsiveness to the different vaccines has been evaluated by different strong statistical analyses. However, in order to validate such findings, it would be extremely relevant to perform a meta-analysis using the same algorithms.

### Data integration

The most challenging aspect of systems biology approaches is the integration of different data types to unveil relationships between genes, transcripts, proteins, metabolites, and epigenetic regulators. To this aim, appropriate analysis and modeling tools are in a continuous progress of development.

Algorithms and software packages designed to integrate heterogeneous data types have been released [132-134] and several publicly available databases of immunology-related transcriptomic datasets have been created in the recent years [135-138]. Furthermore, to improve integration of immunology datasets of these different databases, the Immunological Genome Project initiative has been established recently with the ambitious goal to combine immunology and computational biology laboratories in a systems-level approach [139].

The exploitation of computational methods for data mining and machine learning as well as bioinformatics methods for incorporating prior biological knowledge into data analysis algorithms has been proved to be an effective strategy. Recently, it has been shown the efficacy of applying statistical strategies (i.e. Random Forests method) for integrating genetic (SNPs) and proteomic (cytokine serum concentrations) data collected to elucidate the mechanisms underlying the development of adverse events (AEs) in patients after smallpox vaccination [140]. Combining information from previous studies on AEs related to smallpox vaccination with the genetic and proteomic attributes identified by RF, the Authors were able to build a comprehensive model of AE development that included specific cytokines (i.e. ICAM-1, IL-10, and G-CSF) and a genetic polymorphism in the cytokine gene interleukin-4 (IL4).

Such examples show the efficacy of data integration analysis and it is an easy prediction that in the next future more applications will be reported in the literature.

## Conclusions

The comprehensive analysis at system level of immune response to vaccines and immunotherapies (vaccinomics or systems vaccinology) will provide invaluable knowledge in immunology and genetics which will lead to optimized vaccine development - including the identification of optimal antigens, and antigen formulations (i.e.: adjuvant antigens), inducing the sought cluster of genes and immune pathways leading to the required adaptive immune response. Moreover, it will greatly facilitate screening for responsiveness to vaccines or immunotherapies and understanding eventual failures in individuals enrolled in clinical trials. Indeed, the identification of gene transcriptional profiles or gene polymorphisms closely associated with immune response to such immunological strategies shows the relevance of such comprehensive analysis in the personalization of treatment to obtain the best clinical outcome.

However, the systems biology in vaccinology will go through several steps before becoming a widely used approach. The science is still developing and the complexity and extensive polymorphic nature of immune response genes needs enhanced powerful bio-informatics approaches in order to inexpensively manage the vast mass of genetic information. Moreover, validation studies in larger settings of different genetic background will be required to distinguish between natural and immune-response related genetic - gene expression or polymorphism - modifications. Indeed, only few examples show that genes are modulated in response to vaccination with a cause-effective relationship [141,142]. One way to overcome such problem is to evaluate the results within the context of known pathways or to combine multiple data types. Moreover, according to the vaccine or immunotherapy evaluated, the predictive target cell population should be identified which is not always represented by peripheral blood mononuclear cells.

Nevertheless, regardless the technical, cultural and financial challenges, systems biology applied to vaccinology represents a primary way to go in order to develop novel vaccines and to re-develop established vaccines, switching from the "empirical" to the "knowledge-based" age of vaccinology. This should enable the development of even more successful vaccines for preventive as well as therapeutic intervention strategies for human diseases according to individual- or group-personalized strategy.

## Abbreviations

PRRs: Pathogen recognition receptors; TLRs: Toll-like receptors; HIV: Human Immunodeficiency virus; HCV: Hepatitis C virus; TNFβ: Tumor necrosis factor beta; IL-6: interleukin 6; IL-1β: interleukin 1 beta; IL-12: interleukin 12; APCs: antigen-presenting cells; DCs: dendritic cells; TNFα: Tumor necrosis factor alpha; MIP1α: Macrophage inflammatory protein 1 alpha; MIP1β: Macrophage inflammatory protein 1 beta; PAMS: pathogen-associated molecular signals; MAGE-A3: Melanoma-associated antigen 3; IFN: Interferon; MDDCs: monocyte-derived dendritic cells; PMBCs: peripheral blood mononuclear cells; CCL5: Chemokine (C-C motif) ligand 5; CXCL9: Chemokine (C-X-C motif) ligand9; CXCL10: Chemokine (C-X-C motif) ligand10; SNP: single-nucleotide polymorphism; MyD88: Myeloid differentiation primary response 88; GM-CSF: Granulocyte-macrophage colony-stimulating factor; TOLLIP: Toll interacting protein; TIRAP: toll-interleukin 1 receptor (TIR) domain containing adaptor protein; TICAM1: Toll-like receptor adapter molecule 1

## Competing interests

The authors declare that they have no competing interests.

## Authors' contributions

LB was responsible for the overall planning and coordination of the review as well as writing the paper; EW, MLT, FMB and FM equally contributed to the elaboration of the paper. All authors read and approved the final manuscript.
